# Lallemand’s Operation for Aneurism by Anastomosis

**Published:** 1838-02-28

**Authors:** B. H. Coates


*Lallemand's Operation for Aneurism by Anastomosis.*

[Communicated by Dr. B. H. Coates.]

Philadelphia, Feb. 20th, 1838.

*Gentlemen:*—

I have the pleasure of stating that my success with Professor Lallemand's operation for the cure of an erectile tumour, in the case of Mr. J. S——, appears to be complete. The prominence of the tumour has now nearly disappeared; the parts in the vicinity of the orifices are of a pale pink colour, while a band nearly as pale as the natural colour of the skin extends quite across the original seat of disease, yielding the sensation of but little more hardness than the healthy parts. Hardening is still distinctly felt about the orifices, and thin scabs adhere to most of them. Mr. S. has subjected himself to many of the causes which formerly made the tumour enlarge itself, and which have produced that effect upon the other similar masses with which he is affected; but this has been without result as regards the one on which the operation was performed. I have no doubt that the disease is entirely cured; and am surprised at the rapidity with which, even in his young and healthy constitution, the whole hardened mass is disappearing.

The tumour selected for the first operation was about an inch in diameter, rounded, with slight irregular projections, protruding the skin to about a quarter of an inch, but not adhering to the skin, and leaving that integument of its natural colour. It was situated upon the back part of the arm, a little above the elbow. For several years it had rather increased in size. Eight needles were introduced, and the extremities cut off with a cutting forceps. Each needle was introduced in the manner of acupuncturation, a little to the outside of the tumour, the points issuing upon the opposite side. On the sixth day, a slight erysipelas appeared; being the only material inconvenience experienced till that time from the irritation. As the space surrounding the two needles had suppurated rather freely, these were withdrawn: a flax-seed poultice was applied. Next day the remaining needles were removed; and on the next, all dressings omitted, except a cushion to protect the part from injury.

I have several of these singular productions under my care; and intend, when the number of results is more considerable, to communicate an abstract of them to the public.

The operation above described, was performed in presence of Dr. Wm. H. Brinckle.

I am very respectfully, &c.

B. H. Coates.

Drs. Biddle and Clymer, Editors of the Examiner.

